# Intracellular potassium levels orchestrate circadian rhythmicity and cell division

**DOI:** 10.1038/s41467-026-73351-2

**Published:** 2026-05-22

**Authors:** Sergio Gil Rodríguez, Louise L. Hansen, Olivia J. P. Fraser, Yen Peng Chew, Rebecca K. Spangler, Ellen Grünewald, Andrew D. Beale, Beverley M. Rabbitts, Alessandra Stangherlin, John S. O’Neill, Carrie L. Partch, Priya Crosby, Gerben van Ooijen

**Affiliations:** 1 School of Biological Sciences, University of Edinburgh, Max Born Crescent, Edinburgh, UK; 2 Department of Chemistry and Biochemistry, University of California Santa Cruz, Santa Cruz, CA USA; 3 UKRI MRC Laboratory of Molecular Biology, Francis Crick Ave, Cambridge, UK; 4 University of Cologne, Faculty of Medicine and University Hospital Cologne, Cologne Excellence Cluster for Aging and Aging-Associated Diseases (CECAD), Institute for Mitochondrial Diseases and Ageing, Joseph-Stelzmann-Str, Cologne, Germany; 5 Howard Hughes Medical Institute, University of California Santa Cruz, Santa Cruz, CA USA; 6 Center for Circadian Biology, University of California San Diego, La Jolla, CA USA

**Keywords:** Circadian rhythms, Cell biology

## Abstract

Circadian (~24 h) rhythms are a fundamental feature of life, and their disruption increases the risk of infectious diseases, metabolic disorders, and cancer. We previously identified circadian oscillations in intracellular potassium concentrations in cells across kingdoms. Using highly divergent eukaryotic cell types, we now show that potassium levels act to regulate the period and phase of clock gene expression rhythms, therefore establishing intracellular potassium as a bona fide regulator of cellular circadian rhythms. Intracellular potassium also regulates critical events in the cell cycle. Strikingly, we observe that manipulating potassium levels inhibits cell proliferation in a circadian phase-dependent manner. As the timing of cell division is tuned by the circadian clock, we hypothesised that potassium rhythms could mechanistically link cell proliferation rhythms to the circadian cycle. In line with this hypothesis, we find that potassium levels are not only sufficient to instruct the timing of cell proliferation, but also essential to maintain coherent coupling between circadian rhythms and proliferation rhythms. These results establish circadian potassium rhythms as a primary factor coupling the cell- and circadian cycles in eukaryotic cells.

## Abstract (web summary)

Circadian rhythms are ~24-hour biological cycles that influence processes from immunity to metabolism, and this study explores how intracellular potassium contributes to their regulation. The authors show that potassium levels actively set the period and phase of clock gene rhythms and are essential for coupling circadian timing to cell-division cycles, making potassium an important regulator linking the circadian and cell cycles.

## Introduction

Evolution has provided most eukaryotes with an internal biological timekeeping system to anticipate predictable environmental changes that occur due to Earth’s daily rotation^1^. This endogenous and self-sustaining mechanism, colloquially known as the circadian clock, enhances physiology, metabolism, and overall fitness^1,2^. Conversely, altered or disrupted circadian rhythmicity impacts on many key cellular processes and results in increased risk of disorders and pathologies, including metabolic syndrome and cancer^3–12^. At the cellular level, circadian rhythmicity involves the rhythmic expression of clock genes that engage in Transcriptional/Translational Feedback Loops (TTFLs)^1^, further regulated by highly conserved post-transcriptional mechanisms^13–15^. Circadian rhythms in TTFLs and other cellular properties are commonly modelled using sine waves, with the key features of period (the time it takes for an oscillation to complete), phase (the specific timing of the peaks and troughs of oscillations), and amplitude (the variation of oscillations around the midpoint).

Previously, we reported circadian rhythms in the concentration of intracellular magnesium and potassium in representative species from all eukaryotic kingdoms^16^. These ion rhythms functionally regulate fundamental cellular processes including translation, metabolism, and proteostasis^16–18^. Potassium is the most abundant ion in any eukaryotic cell, constituting an average of 0.2% of the total body weight in humans^19^ and 2-10% of dry weight in plants^20^, and acts to maintain fluid and electrolyte balance over membranes^21^. In humans, even small alterations in the intracellular flux of potassium are linked to metabolic disorders and cancer^17,22^. High-amplitude circadian rhythms in potassium are therefore likely to impact upon crucial cellular processes in any eukaryote, such as membrane potential, ion homeostasis, enzyme activity, and osmotic balance^16,23,24^. Most notably for this work, intracellular potassium and potassium channels are well-established to be one of several mechanisms that regulate appropriate progression of the cell cycle^25,26^, and previous indications exist that potassium transport inhibition suppresses proliferation of cancer cells^27–30^.

Bidirectional interaction between the circadian clock and the cell cycle has been described across organisms, from cyanobacteria to mammals^31–35^. The term ‘coupling’ is used to refer to phase and period synchrony between the circadian clock and the cell cycle. The relative strength of coupling can differ between organisms: in the unicellular marine alga *Ostreococcus tauri*, cytokinesis is strictly limited to a specific time of day^36^, while most mammalian systems show more flexible coupling between these two biological cycles^31^. Cues that synchronise circadian rhythms of mammalian cells with the external day/night cycle also shift timing of cell division and growth, indicating a preferred circadian time for cell cycle events^37–40^. Mechanistically, previous work has shown coupling between the mammalian cell cycle and circadian rhythms to involve circadian regulation of cell cycle regulators such as Wee1 kinase and cyclin B1-Cdc kinase by the mammalian TTFL^33,39–41^. In organisms that lack homologues of the core mammalian TTFL components, the mechanistic connection between the circadian system and the cell cycle has, to the best of our knowledge, been almost entirely unexplored.

In this study, we tested the hypotheses that circadian rhythms in potassium are a regulator of both timekeeping and cell proliferation individually, and also act to couple these two rhythmic processes. As both potassium rhythms and circadian-cell cycle coupling are observed across eukaryotes, we posited that this regulation of circadian and cell cycle progression by potassium would be highly conserved. We thus employed two of the most divergent cell types among established circadian models: mouse fibroblasts and the marine alga, *Ostreococcus tauri*. These two organisms are separated by ~1 billion years of evolution^42^ and representative of different kingdoms of eukaryotes (mammals and plants, respectively). Reflecting their evolutionary divergence, the two organisms have distinct circadian TTFL protein machinery, with almost no protein sequence or structural homology. The mouse TTFL consists of the heterodimeric transcription factor CLOCK:BMAL1, which drives expression of the circadian repressors, PERIOD (PER) and CRYPTOCHROME^43^. In contrast, the *Ostreococcus* clock is based on a plant-like feedback loop between the transcription factors CCA1 and TOC1^14,16^. In both mammals and *Ostreococcus*, these core circadian transcriptional regulators are commonly termed ‘clock genes’. In addition to their distinct circadian TTFLs, these cell types are adapted to disparate extracellular conditions: while the tiny (1-2 µM) cells of *Ostreococcus* cells are suspended in sea water, mammalian cells (10-30 µm) live in a complex multicellular environment where the concentration of potassium and other biologically relevant ions is homeostatically controlled. Our comparative approach therefore provides an enviable platform to probe the function of potassium rhythms across eukaryotic cell types. We provide evidence that potassium rhythms closely interact with TTFL rhythmicity and cell proliferation, and present a general model for the coupling of the eukaryotic cell and circadian cycles.

## Results

### Direct reciprocal feedback between potassium rhythms and clock gene expression

We have previously shown in cells from across biological kingdoms that intracellular potassium levels oscillate with a circadian rhythm and phase similar to magnesium, peaking in the subjective night^16^. This also holds true for *Ostreococcus* (Supplementary Fig. 1a). As in other species^19,20^, potassium is the most abundant intracellular cation in *Ostreococcus*, with a large gradient over the plasma membrane (Supplementary Fig. 1b, c). This gradient produces corresponding rhythms in membrane capacitance over the circadian day in *Ostreococcus* (Supplementary Fig. 1d), as it does in human red blood cells^17^. To enable us to manipulate intracellular potassium levels in *Ostreococcus*, we tested a set of treatments that have been shown to affect intracellular potassium concentrations, or [K^+^]_i_, in other species^44,45^. In line with previous studies, changing the concentration of extracellular potassium, [K^+^]_e_, induced corresponding changes in [K^+^]_i_ (Supplementary Fig. 1e). [K^+^]_i_ could also be lowered using either caesium (Cs^+^, a non-biological ion that competes with potassium for the same transport machinery) or the voltage-dependent potassium ion channel inhibitor 4-aminopyridine (4-AP). These experiments define a set of tools to manipulate intracellular potassium levels across eukaryotic cell types.

**Fig. 1:**
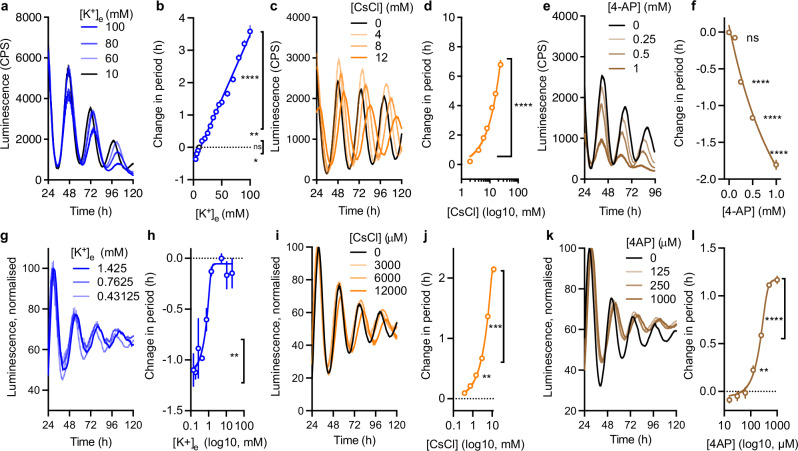
Intracellular potassium levels regulate circadian gene expression. **a**–**f** Contain data from *Ostreococcus* expressing the CCA1-LUC circadian reporter, and **g–l** contain data from mouse expressing the PER2-LUC circadian reporter. **a**, **b** Changes in circadian period of *Ostreococcus* at different extracellular potassium concentrations, as example traces (**a**) and a dose response curve (**b**). n = 16, mean±SEM, one-way ANOVA, Dunnett’s multiple comparisons test vs. 10 mM K^+^ control. **c**, **d** Example traces (**c**) and dose response curve (**d**) for the effect of caesium on circadian period in *Ostreococcus*. n = 8, mean±SEM, one-way ANOVA, Dunnett’s multiple comparisons test vs. mock treated controls. **e**, **f** Example traces (**e**) and dose-response curve (**f**) for the effect of 4-AP on circadian period in *Ostreococcus*. n = 16, mean±SEM, one-way ANOVA, Dunnett’s multiple comparisons test vs. mock. **g**, **h** Example traces (**g**) and dose response curve (**h**) of confluent PER2-LUC fibroblasts treated with increasing concentrations of extracellular potassium. n = 3 or 2 (see source data), mean ± SEM, one-way ANOVA, Dunnett’s multiple comparisons test vs. 5.4 mM K^+^ control. **i**, **j** Example traces (**i**) and dose response curve (**j**) of confluent PER2-LUC fibroblasts treated with increasing concentrations of CsCl show increasing circadian period. n = 3, mean ± SEM, one-way ANOVA, Dunnett’s multiple comparisons test vs. mock. **k**, **l** Example traces (**k**) and dose response curve (**l**) of confluent PER2-LUC fibroblasts treated with increasing concentrations of 4-AP show increasing circadian period. n = 3 or 2 (see source data), mean ± SEM, one-way ANOVA, Dunnett’s multiple comparisons test vs. mock.

To investigate reciprocal feedback between TTFL rhythmicity and potassium rhythms in *Ostreococcus*, we characterised the effect of [K^+^]_i_ on clock gene expression rhythms using the in vivo circadian reporter CCA1-LUCIFERASE (CCA1-LUC)^46^. Under constant light conditions (LL), increasing [K^+^]_e_ dose-dependently induced long period clock gene rhythmicity compared to the control [K^+^]_e_ of 10 mM (Fig. 1a, b). Likewise, caesium dose-dependently induced period lengthening (Fig. 1c, d). To verify that period lengthening by caesium is indeed caused by direct competition with potassium, we tested the efficacy in the presence of varying concentrations of potassium. Indeed, the effect of caesium can be modulated by changing extracellular potassium (Supplementary Fig. 1f, g). Unlike caesium, 4-AP induced short period clock gene rhythms (Fig. 1e, f). This discrepancy is likely due to their different modes of action^47^. As treatments with 4-AP and extreme [K^+^]_e_ led to reduced amplitude rhythms, we verified cell viability under these treatments. Upon release of treatments, cells rapidly resume rhythmicity (Supplementary Fig. 1h) and flow cytometry analyses did not suggest loss of cell integrity after 120 h of treatments (Supplementary Fig. 1i), confirming that these treatments do not substantially impact cell viability. The observed effects on circadian period are specific to changes in potassium and not induced by a change in extracellular salinity, as clock gene expression in *Ostreococcus* is resilient to changes in overall salinity beyond the range used in this study (Supplementary Fig. 1j).

To test whether the same direct and dose-dependent effect of potassium levels is observed on the mammalian TTFL system, we repeated these treatments using lung fibroblasts derived from the PERIOD2-LUCIFERASE (PER2-LUC) mouse^48^. As in *Ostreococcus*, decreasing potassium levels shortened the period of circadian gene expression (Fig. 1g-h). However, unlike the marine cells of *Ostreococcus*, mammalian cells are highly sensitive to changes in extracellular osmolarity. To overcome this, osmolarity of culture media was kept constant at 350 mOsm/kg across experiments, and therefore only a small range of [K^+^]_e_ was experimentally testable. We also tested the effects of 4-AP and caesium, both of which dose-dependently increased circadian period in both confluent (Fig. 1i-l) and actively dividing PER2-LUC fibroblasts (Supplementary Fig. 1k–n). We note that 4-AP produced different period effects in *Ostreococcus* versus mouse cells. *Ostreococcus* is a species of unicellular algae that grows freely in the ocean with an osmolarity of approximately 1000 mOsm/kg^49^, while mammalian cells experience ~350 mOsm/kg in plasma. Our observations that 4-AP induces short period in *Ostreococcus* and long period in fibroblasts is likely indicative of this vastly different driving force for ionic movement, which likely produces differences in relative expression and utilisation of potassium channels.

Together, the results in Fig. 1 reveal a direct effect of potassium levels on the period of eukaryotic clock gene expression rhythms.

### Potassium levels affect timekeeping in a circadian phase-dependent manner

**Fig. 2:**
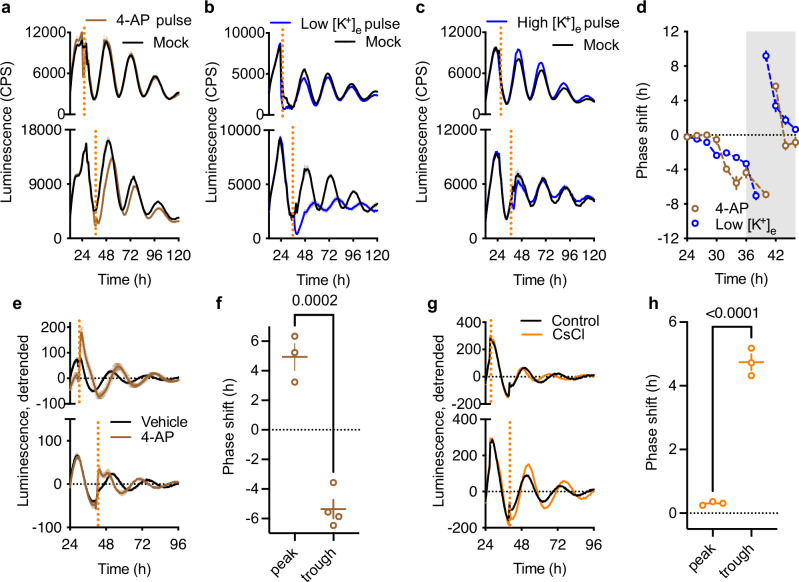
Potassium affects the phase of clock gene expression in a time-of-day dependent manner. **a**–**d** Contain data obtained from *Ostreococcus* expressing CCA1-LUC, **e**–**h** contain data from murine primary fibroblasts expressing PER2-LUC. **a**–**c** Pulsed treatments (2 h) with 4-AP (**a**), low extracellular potassium (**b**) or high extracellular potassium (**c**) at subjective dawn (top panels) or dusk (bottom panels) differentially affect the subsequent phase of the CCA1-LUC marker in *Ostreococcus*. n ≥ 8, mean ± SEM, treatment times indicated by vertical orange dotted lines. **d** Phase response curves from *Ostreococcus* pulse treated with low extracellular potassium (blue) or saturating concentrations of 4-AP (brown). Phase changes relative to control treated wells. Grey area represents the subjective night. n = 8 to 12 (see source data), mean±SEM. **e**, **f** PER2-LUC traces (**e**) and phase-shift (**f**) from mouse fibroblasts treated with 4-AP at the peak or trough of PER2 expression. n = 3 or 4 (see source data), mean ± SEM, two-sided unpaired t-test. **g**, **h** PER2-LUC traces (**g**) and phase-shift (**h**) from mouse fibroblasts treated with CsCl at the peak or trough of PER2 expression. n = 3, mean ± SEM, unpaired t-test. NaCl was used as a control for CsCl.

Next, we tested whether potassium affects clock gene expression in a circadian phase-dependent manner. We subjected *Ostreococcus* cells to 2 h pulse treatments (applied, then washed off) with 4-AP. We compared treatments at subjective dawn (the phase of high intracellular potassium) to treatments at subjective dusk (low intracellular potassium). Treatments with 4-AP at subjective dawn had no effect on clock gene expression compared to controls, while treatments at subjective dusk produced a clear shift in circadian phase (Fig. 2a). Similarly, we found that circadian gene expression was only sensitive to a treatment pulse with low extracellular potassium at subjective dusk and not dawn (Fig. 2b). The corresponding treatments with high potassium did not induce phase shifts (Fig. 2c). We then analysed the circadian phase of cells treated with low potassium or 4-AP compared to mock treatments over a full 24 h cycle. A striking difference in phase sensitivity is observed over the 24 h cycle, with the greatest phase shifts observed during the early subjective night (Fig. 2d). We verified that these phase effects are not related to differential effects on circadian period, as no period effects are observed with these transient treatments at any phase (Supplementary Fig. 2a).

We next asked whether modulating clock gene expression had a reciprocal effect on potassium rhythms. In cells that are photosensitive, such as *Ostreococcus*, changing the fluence rate of light affects clock gene expression rhythms^50,51^. We observed dampened CCA1-LUC rhythms that oscillated at a shorter period and advanced phase under high light levels versus the standard dim light levels (Supplementary Fig. 2b, c). Interestingly, potassium rhythms also oscillated at an advanced phase and faster period under high versus dim light (Supplementary Fig. 2d), highlighting a close connection between the parameters of circadian potassium oscillations and clock gene expression rhythms.

We subsequently tested the phase dependency of mammalian clock gene expression in response to changes in potassium levels. As media change with serum is a phase-resetting cue for mammalian cells in culture^52^, it was not possible to test the effects of acute low [K^+^]_e_. We therefore tested phase sensitivity to 4-AP or caesium treatment. Treatments were applied in fibroblasts at the peak of PER2 expression (the phase of high intracellular potassium^18^) versus the trough of PER2 expression (low intracellular potassium). While 4-AP induced phase shifts at both timepoints, it did so in strikingly opposite directions (Fig. 2e, f). Caesium treatment at the peak of PER2 expression did not affect TTFL phase, but clearly delayed circadian phase compared to controls at the trough of PER2 (Fig. 2g, h).

Combined, the results in Fig. 2 indicate that potassium levels regulate circadian gene expression in a phase-dependent manner. As potassium abundance is circadian-regulated^16,17^ and feeds back to regulate the period (Fig. 1) and phase (Fig. 2) of clock gene expression rhythms, we propose [K^+^]_i_ as a conserved regulator of cellular circadian rhythmicity.

### Potassium levels affect cell proliferation in a circadian phase-dependent manner

**Fig. 3:**
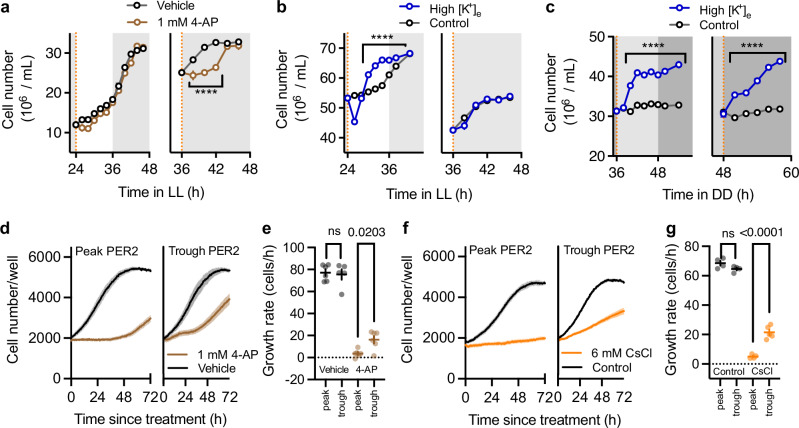
Potassium affects cell proliferation in a time-of-day dependent manner. Data in (**a**–**c**) was obtained in *Ostreococcus* cells, and data in panels **d–g** was obtained in mouse fibroblasts expressing PER2-LUC. **a**, **b** *Ostreococcus* cell number upon 2 h pulsed treatments with 4-AP (**a**) or high potassium (**b**) at subjective dawn (left panels) versus subjective dusk (right panels) under constant light conditions. **c** As **b**, but under constant darkness. In (**a**–**c**), darker areas represent subjective night and lighter areas subjective day; orange dotted lines indicate treatment times. n ≥ 5, mean±SEM, two-way ANOVA, Dunnett’s multiple comparisons test vs. control. **d** Treatment of actively dividing PER2-LUC fibroblasts with 1 mM 4-AP at the peak (left panel) or trough (right panel) of PER2 expression results in differing effects on cell proliferation. n = 6 or 5 (see source data), mean ± SEM. **e** Quantification of log-phase proliferation rate from treatments in (**d**). Two-sided Šidák’s multiple comparison’s test. **f** Treatment of actively dividing PER2-LUC fibroblasts with 6 mM CsCl at the peak (left panel) or trough phase (right panel) of PER2 expression. n = 3 to 5 (see source data), mean ± SEM. **g** Quantification of log-phase proliferation rate from treatments in (**f**). Two-sided Šidák’s multiple comparison’s test.

In mammalian cells, cell size and cell proliferation rates are highly sensitive to intracellular potassium levels^25,26^. We first verified that potassium levels have the same effect on cell size and proliferation in *Ostreococcus* (Supplementary Fig. 3a-d). We next hypothesised that if potassium rhythms interact closely with both circadian gene expression rhythms (Figs. 1 and 2) and key properties of cell proliferation, they could be a primary factor mechanistically coupling circadian timing to proliferation rhythms in eukaryotes. *Ostreococcus* is a particularly suitable model cell to test this hypothesis, as the *Ostreococcus* cell cycle is tightly gated by the circadian clock^36^ with a predominant phase of cell division in the early night^32^. We found that light fluence rates, which affect both clock gene expression and potassium rhythms (Supplementary Fig. 2b-d) also affected cell size and proliferation (Supplementary Fig. 3e-f), providing further circumstantial evidence for a tight interaction between TTFL and potassium rhythms and cell proliferation. *Ostreococcus* cells were then subjected to 2 h pulsed treatments of 4-AP at subjective dawn versus dusk, with cell proliferation monitored through manual counting of cells. 4-AP treatment at subjective dawn (when intracellular potassium levels are already low) had no effect on the phase of cell proliferation but induced a large delay when applied at subjective dusk (Fig. 3a). Conversely, pulsed treatments with high [K^+^]_e_ at subjective dusk had no effect on the subsequent proliferation pattern, while at dawn, a large phase advance in proliferation was observed (Fig. 3b). Finally, a pulse of low [K^+^]_e_ did not greatly affect cell proliferation when applied at subjective dusk, but induced cell death and an arrest of cell proliferation at subjective dawn (Supplementary Fig. 3g). These results highlight that modulating [K^+^]_i_ is sufficient to advance or delay the timing of proliferation with respect to prior circadian phase.

Interestingly, strong effects of low [K^+^]_e_ on *Ostreococcus* cell proliferation at subjective dawn (Supplementary Fig. 3g) occurred at the opposite circadian phase as the effect of the same treatment on clock gene rhythmicity (Fig. 2b), and the effects of high [K^+^]_e_ on cell proliferation at dawn (Fig. 3b) happened in spite of the same treatment not affecting transcriptional rhythms at all (Fig. 2c). These observations indicate that potassium gradients affect cell proliferation and TTFL rhythmicity separately. We therefore asked whether potassium could also induce changes in the cell cycle in the absence of TTFL rhythms. A unique aspect of the cell biology of *Ostreococcus* is that constant darkness induces a lack of transcriptional rhythms^14^ as well as a loss of rhythmicity of intracellular potassium levels^16^. We found that cells did not proliferate under darkness (Fig. 3c), meaning that under these conditions all three rhythmic cogs of TTFL, potassium rhythms, and cell cycle rhythms are arrhythmic. However, pulsed treatments with either high (Fig. 3c) or low potassium (Supplementary Fig. 3h) led to a rapid and strong increase in cell proliferation regardless of whether treatment time was at subjective dawn or dusk. We verified that these treatments did not restore any TTFL rhythms under darkness (Supplementary Fig. 3i). Our results therefore establish that in the absence of cellular rhythmicity, a sudden enforced potassium gradient is sufficient to instruct cell proliferation. The loss of differential effects between dawn and dusk indicates that a functional TTFL is likely required to deliver the phase sensitivity normally observed under light-dark or constant light conditions.

Direct coupling between the period of circadian and cell cycle rhythms has also previously been demonstrated in freely proliferating mammalian cells^39,40^. To determine whether manipulation of potassium also affects cell proliferation in a circadian phase-dependent manner in mammalian cells, PER2-LUC fibroblasts were subjected to treatment with 4-AP (Fig. 3d-e) or caesium (Fig. 3f-g) at peak versus trough PER2 levels (Supplementary Fig. 3j-k). Unlike in *Ostreococcus*, proliferation of fibroblasts could be precisely measured using quantification of cell number from continuous single-cell imaging. Although cell proliferation rate was reduced by treatments at either phase, treatments at peak PER2 levels (the phase of high intracellular potassium) inhibited proliferation to a greater extent. Notably, as in *Ostreococcus*, the maximal effects of these treatments on cell proliferation and on TTFL rhythmicity (Fig. 2e-f) occurred at opposite circadian phases. This indicates that in both cell types, the effects of intracellular potassium on TTFL and cell proliferation rhythms are distinct.

Overall, the results in Fig. 3 establish that modulating potassium affects eukaryotic cell proliferation in a circadian phase-dependent manner and that, at least in *Ostreococcus*, potassium gradients are sufficient to instruct cell division.

### Manipulating intracellular potassium levels disrupts coupling between the cell and circadian cycle

**Fig. 4:**
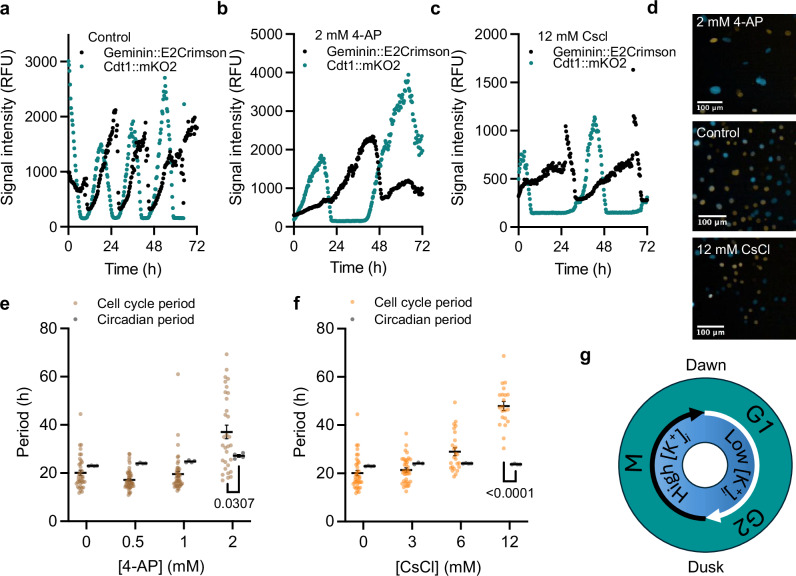
Potassium maintains coupling between circadian gene expression and cell proliferation. This figure only contains data obtained from the standard mouse fibroblast cell line, NIH 3T3. **a**–**c** Representative single-cell data from unsynchronised NIH 3T3 cells expressing the FUCCI−2A system under control conditions (**a**) and treated with 2 mM 4-AP (**b**) or 12 mM CsCl (**c**). **d** Representative images of NIH 3T3 FUCCI−2A cells treated with 2 mM 4-AP, 12 mM CsCl, or control. **e**, **f** Quantification of the change in period of the circadian marker Per2:LUC (black data points, n = 4, mean ± SEM) versus the cell cycle marker Cdt1::mKO2 (coloured data points) under increasing concentrations of 4-AP (**e**) or CsCl (**f**). For cell cycle period, n = 21 to 38 (see source data), individual nuclei were quantified from at least 3 independent wells, mean ± SEM, two-sided two-way ANOVA with Fisher’s LSD test. **g** A general model for the relationship between potassium rhythms and cell division in eukaryotic cells. Likely timing of cell cycle stages and potassium rhythms plotted on a 24 h clockface. White arrow denotes day, dark arrow denotes night.

We posited that if intracellular potassium mechanistically coupled the two cyclical processes of circadian gene expression and cell cycle progression, then manipulating [K^+^]_i_ should lead to a loss of coupling, such that the two processes cease to run in parallel. While circumstantial evidence in that direction is provided by the opposite phases of sensitivity to potassium-related treatments between transcriptional rhythms and cell proliferation, the tools to test this directly are not available in *Ostreococcus*. We therefore employed the mouse fibroblast cell line NIH 3T3 expressing the FUCCI-2A system, which allows for non-invasive imaging of cell cycle progression through expression of fluorescently-tagged fragments of Cdt1 (accumulating in G1) and Geminin (accumulating in S/G2/M)^53^. As in primary PER2-LUC mouse lung fibroblasts (Figs. 1–3), 4–AP and caesium lengthened the period of gene expression rhythms in actively dividing NIH 3T3 cells (Supplementary Fig. 4a-d).

We subjected circadian phase-unsynchronised, rapidly dividing NIH 3T3 FUCCI-2A cells to single-cell imaging (Supplementary Movie 1-3). Under control conditions, cell division rhythms oscillated with a period in the circadian range (Fig. 4a). This conformed to previous work that demonstrated coupling between the circadian and cell cycles in these cells under these conditions^40^. Upon treatment with 4-AP (Fig. 4b) or CsCl (Fig. 4c) a significant and substantial increase in the average cell cycle period is observed. We verified that no substantially aberrant nuclear morphology was observed, indicating the observed effects are not associated with poor cell health (Fig. 4d and Supplementary Fig. 4e-h). The effects on cell cycle period were dose-dependent (Fig. 4e-f, coloured data points) and accompanied by a decreased rate of proliferation (Supplementary Fig. 4i-l) and an increased proclivity towards cell cycle arrest (Supplementary Fig. 4m-n). No significant difference was observed in the relative timing of cell cycle phases (Supplementary Fig. 4o). Critically, the dose-dependent increases in cell cycle period were much larger than, and did not correlate with, the observed increases in circadian period of clock gene expression rhythms measured in parallel using NIH 3T3 cells expressing Per2-LUC (Fig. 4e-f, black data points). The disparate effects of the same treatments on the periods of clock gene expression versus cell cycle rhythms suggests that modulation of potassium rhythms can disrupt the coupling between these cyclical processes.

The results in Fig. 4 are therefore consistent with our prediction that disrupting intracellular potassium levels leads to a loss of coupling between the circadian and cell cycles.

## Discussion

Combined, the data presented in this paper demonstrate a clear role, conserved across eukaryotic cell types, for rhythmic intracellular potassium levels in orchestrating key rhythmic properties of a cell: clock gene expression and cell division. Furthermore, this work supports a general model where intracellular potassium levels act as a coupling mechanism between the circadian TTFL and cell proliferation rhythms in eukaryotic cells. In this model, cell division is most likely to happen close to the peak of intracellular potassium rhythms (Fig. 4g).

As intracellular potassium is the most abundant intracellular cation, the osmotic consequences of oscillations in potassium cannot be separated from concurrent electrochemical changes over the circadian cycle, as evidenced by electrophysiological results in this paper (Supplementary Fig. 1) and previous work^17^. From known concentrations of intracellular ions at the peak and trough of potassium levels, we have calculated the difference in Resting Membrane Potential (RMP) over the circadian cycle (Supplementary Fig. 4p) and found these to be minor (2 mV for *Ostreococcus* and 5 mV for mammalian cells). While we do not discount the possibility that changes in RMP make a functional contribution, there is no precedent for such modest changes in RMP to affect cell division. We therefore posit that circadian rhythms in potassium specifically act as a primary factor coupling circadian rhythmicity to the cell cycle.

Previous studies have largely focused on direct control of cell division by the transcriptionally-driven circadian TTFL, the exact identity of which varies between eukaryotes. However, an increasing body of knowledge demonstrates robust, evolutionarily-conserved circadian rhythms in post-transcriptional processes that continue without transcription^13,14,16,54^. These observations demonstrate a necessity to expand the focus beyond the TTFL to unravel the links between circadian rhythms and the cell cycle. Intracellular potassium rhythms constitute one example of cellular rhythmicity not fully explained by TTFL rhythmicity, as they are sustained in anucleate cells (human red blood cells)^17^. Critically, our work demonstrates that TTFL and potassium rhythmicity is closely intertwined; for example, changing light levels in *Ostreococcus* similarly affects both. Establishing the causal relationships between TTFL rhythms and potassium rhythms is hampered by our very limited understanding of the identity and mechanisms of the non-transcriptional timekeeping system in eukaryotes. This study highlights the importance of addressing that knowledge gap going forward, as critical physiological properties such as cell proliferation are regulated by it.

A second interesting avenue for future investigations is to consider the functional consequences of potassium rhythms for the rate of protein synthesis and organelle duplication ahead of cell division. Our previous work^18^ showed that circadian rhythms in intracellular ion concentrations buffer intracellular osmolarity in response to daily rhythms in protein synthesis and abundance in confluent cells. Considering this, it is tempting to postulate that in proliferating cells, similar mechanisms are required to regulate cell volume changes in response to protein synthesis during G1 phase^55,56^. In line with this, G1 phase sees a dramatic reduction in [K^+^]_i_ (Fig. 4g), when protein synthesis rates are highest. The increase in potassium during G2 phase, and the associated increase in intracellular osmolarity, is likely to contribute to the ingress of water required for cell growth ahead of cell division.

From a biomedical perspective, alterations in the intracellular balance and flux of potassium are linked to metabolic and cell cycle disorders, including cancer^17,22^. Interestingly, abnormal proliferation phenotypes and cell properties found in multiple cancer cells including glioma, hepatoblastoma, breast cancer or malignant astrocytoma are correlated with altered K^+^ homeostasis or the altered polarization profiles that this causes^27–30,57–59^. Like magnesium rhythms^60^, potassium rhythms are presumably generated by circadian regulation of the activity rather than abundance of the proteins that make up the potassium transport machinery, as no evidence is found for circadian abundance of these proteins in a circadian proteome of *Ostreococcus*^32^. It is therefore interesting to note that the inhibition of K^+^ transporters in tumour cells shows a strong therapeutic potential in cancer treatments, highlighting this as a target for cancer research^57,61^. Additionally, disruption of circadian rhythms is permissive for the development of cancer^9^, and around 50% of cancers show dysregulation of the circadian TTFL. We thus anticipate that the novel fundamental insights reported here into the integration of potassium rhythms, TTFL rhythmicity, and cell proliferation will inform research into the underlying molecular regulation of these coupled systems, ultimately contributing to future cancer research and therapy.

## Methods

### *Ostreococcus tauri* methods

#### Bioluminescence recording

Transgenic CCA1-LUC *Ostreococcus* cells^46^ were grown in vented tissue culture flasks (Sarstedt) at 20 °C in artificial sea water (ASW) with a light intensity of 17 μmol m^−2^ s^−1^ under blue light filters (183 Moonlight Blue Filter, Lee filters). Unless otherwise stated, all experiments were carried out in ASW with the standard 10 mM potassium (full composition of ASW: NaCl 325 mM, MgCl_2_ 50 mM, Na_2_SO_4_ 28 mM, CaCl_2_ 10 mM, KCl 10 mM, NaHCO_3_ 2.4 mM, KBr 0.2 mM, H_3_BO_3_ 0.4 mM, NaF 0.1 mM; supplemented with Guillard’s F/2 marine enrichment solution and 10 nM H_2_SeO_3_. The salinity was adjusted to 30 ppt). Cells were entrained under 12 h light/12 h dark (LD) cycles for 6-7 days to reach optimal cell density for experimental use. CCA1-LUC cell cultures were diluted 1:3 in fresh ASW 30-35 ppt and supplemented with 0.2 mM D-luciferin. 90 µL was added to wells of a 384-well plate (Greiner) and imaged in a luminescence plate reader (Berthold TriStar2) under 2 μmol m^−2^ s^−1^ blue light (183 Moonlight Blue Filter, Lee filters) for 5-7 days under constant light conditions. 4-AP (Sigma) or CsCl (Sigma) treatments were added at 10x concentration. For washout treatments, media from each well was carefully removed and replaced with media containing the appropriate treatment and luciferin, avoiding disturbing the cell aggregates. After the pulse treatments, cells were fully resuspended with fresh ASW media + luciferin. Treatments were performed on the second day of constant conditions (24 h into LL or 36 h into DD) and all fall within a range of overall salinity of 30-35 ppt. Salinity experiments were performed by adjusting [NaCl]. For differential light conditions, light intensity was adjusted at the start of imaging. High light = 17 μmol m^−2^ s^−1^ and dim light = 2 μmol m^−2^ s^−1^. Results were analysed and plotted using GraphPad Prism v9. Period and phase analyses were performed using BioDare2^62^ as previously published^32^.

#### Cell number and area analysis

For cell number and cell area analyses, *Ostreococcus* cells subjected to identical experimental settings and conditions as for the luminescence assays were harvested and counted using a haemocytometer with a light microscope on 40x magnification. Data was collected from at least 5 technical replicates for each time point. For cell area, cell samples were quickly mixed 1:1 with -80 °C methanol (70%) to preserve cell volume. Pictures were taken with a light microscope from at least 100 cells for each condition and cell area was measured with ImageJ. For flow cytometry analyses of cell health after 120 h of treatments with differential [K^+^]_e_ or [4-AP], 10,000 cells were analysed per treatment using BD FACSCanto II (BD Biosciences, San Jose, CA, USA) with excitation laser (488 nm) and detected using fluorescent channel (660 ± 20 nm) for chlorophyll autofluorescence. BDFacs Diva was used for data acquisition and FCS Express 7 for analysis. Gates were drawn on control samples using forward and side scatter. A further gate was drawn to determine chlorophyll autofluorescence, and ‘normal cells’ were defined as the percentage of chlorophyll positive cells within sizing gate.

#### Ion analysis

For ion analyses, 25–30 ml *Ostreococcus* cell cultures were collected at stated times, pelleted, and washed twice with 1 M Sorbitol (Sigma) to remove all salts present in the media. Pellets were then resuspended in 100 µL of 69% Nitric acid (MERCK) and digested O/N at RT. Samples were diluted to 5% Nitric acid with HPLC grade water. 4 technical replicates for each time point were analysed using Microwave Plasma - Atomic Emission Spectrometry (MP-AES 4210, Agilent) as reported previously^24^. Data were corrected for cell counts throughout the timeseries.

#### Dielectrophoresis

For dielectrophoresis (DEP), *Ostreococcus* cultures were entrained under 14 h light:10 h dark cycles before transfer into constant conditions. Starting at 24 h, every 4 h, 15 mL of 27.5 ×10^6^ cells/mL cultures were transferred to 15 mL falcon tube and washed twice in 15 mL iso-osmotic 1 M sorbitol by centrifuging for 2 min at 4472 *g*, and then resuspended in a final volume of 0.5 mL 1 M sorbitol. 75 µL of these cell suspensions were pipetted into 3DEP chips (DEPtech, Heathfield, UK), which were subsequently inserted into a 3DEP reader (DEPtech). Pin connections energised each well at 10 Vp–p, with a different frequency applied to each of the 20 wells and with the wells collectively energised for 10 s at five points per decade (10 kHz—20 MHz). This was repeated at each time point at least three times. The raw data were fitted with a single-shell model in order to extract the electrophysiological parameters as previously^17,63^, accepting spectra producing R^2^ values of 0.9 or greater.

### Mammalian cell methods

#### Isolation of cell lines

NIH 3T3 Rev-Erbα-Venus FUCCI-2A cells were a gift from Franck Delaunay. NIH 3T3 Per2:LUC cells were generated previously^64^. PERIOD2-LUCIFERASE^48^ lung fibroblasts were derived from mouse lung kindly donated by David Welsh. Mouse primary fibroblasts were isolated according to an established protocol^65^. For this, lung tissue was stored in ice-cold PBS for up to 24 hrs. Tissue samples were subsequently removed from PBS and cut in to ∼1 mm^3^ sections using a pair of sterile scalpels, before being transferred to a 50 mL falcon tube with 10 mL “digestion medium” (DMEM/F12 supplemented with pen/strep, Mycozap Plus PR and 0.14 U/mL Liberase) and incubated at 37 °C, stirring slowly, for 30 min, or until the tissue fragments turned white. The tissue fragments were then titurated using a 10 mL pipette and 40 mL “initial culture medium” (DMEM/F12, supplemented with pen/strep, Mycozap Plus PR and 15% HyClone FetalClone III) added before the tube was centrifuged at 700x g for 5 min. The resulting supernatant was discarded, the pellet resuspended in a further 20 mL “initial culture media” and the tube centrifuged for a further 5 min. The supernatant was again discarded, the pellet resuspended in 10 mL “initial culture media” and transferred to a 10 cm tissue culture dish and incubated at 37 °C, 5% CO_2_, 3% O_2_. After 7 days, media was refreshed and after a further 7 days, cells were split and re-plated in “selection medium” (MEM supplemented with pen/strep, non-essential amino acids, sodium pyruvate and 10% HyClone FetalClone III). After a further 2 weeks, cells were transferred to DMEM-based culture medium (DMEM supplemented with pen/strep and 10% HyClone FetalClone III). Immortalization was achieved by serial passage of cells at 37 °C, 5% CO_2_, 20% O_2_. Cell lines were authenticated by observation of morphology and by continued expression of the bioluminescent reporter.

#### Bioluminescence recording

For bioluminescence assays in confluent monolayers, cells were grown to confluence in 12 well or 35 mm dishes in high-glucose (27.8 mM), glutamax-containing DMEM (GIBCO) supplemented with 10% serum (HyClone FetalClone III, Themofisher) and pen/strep and subjected to temperature cycles of 12 h 37 °C followed by 12 h at 32 °C. Confluent cultures were kept for up to 4 weeks with media refreshed every 7-10 days. For bioluminescence assays in proliferating cells, cells were seeded at ~7% confluency in the same conditions and kept for 36 h. For recording, cells were changed to MOPS-buffered “Air Media” (Bicarbonate-free DMEM, 5 mg/mL glucose, 0.35 mg/mL sodium bicarbonate, 0.02 M MOPS, 100 μg/mL pen/strep, 1% Glutamax, 1 mM luciferin, pH 7.4, 325 mOsm)^66^. 10% serum was used in all cases except for low [K]_e_ experiments. Cells were then transferred to a Lumicyle (Actimetrics) or an ALLIGATOR (Cairn Research), where bioluminescent activity was recorded at 15 min intervals using an electron multiplying charge-coupled device (EM-CCD) at constant 37 °C. For treatment, cells were removed from recording on a heatpad and kept at constant 37 °C for treatment before returning to recording. Detrending of bioluminescent traces, where appropriate, was performed using a 24 h moving average detrend. Bioluminescent traces from mammalian cells were fitted with damped cosine waves in Prism 10 (GraphPad) using Eq. 1:

1 $$y={mx}+c+{{{\rm{Amplitude}}}}\cdot {{{\rm{e}}}}-{kx}\cdot \cos (2{{{\rm{\pi }}}}({{{\rm{x}}}}-{{{\rm{phase}}}}){{{\rm{period}}}})$$

where y is the signal, x the corresponding time, amplitude is the height of the peak of the waveform above the trend line, k is the decay constant (such that 1/k is the half-life), phase is the shift relative to a cos wave and the period is the time taken for a complete cycle to occur.

#### Fluorescence imaging and analysis

For fluorescence cell imaging of Rev-Erbα-Venus FUCCI-2A NIH 3T3s, cells were plated to ~7% confluency in a black, clear-bottomed 96-well plate in MOPS-buffered ‘Air media’ with 10% serum but lacking luciferin (see above) with the indicated concentrations of CsCl, NaCl or 4-AP. Cells were then maintained at constant 37 °C for 36 h before moving to an Opera Phenix Plus (Revvity Perkin Elmer, at the UCSC Chemical Screening Center RRID SCR_021114)) for recording at constant 37 °C in a humidified, dark environment. Cells were imaged every 18 min with 9 fields of view per well, using a 10x air objective, two peak focusing at the -5 um focal plane, 50 um pinhole spinning disc, 2160×2160 px camera, binning 2, and the following channels: brightfield with transmitted light and a 650-760 nm filter with 20 ms exposure at 20% light power; mKO2 with a 561 nm excitation laser and a 571-596 nm emission filter with 60 ms exposure at 95% laser power; and E2-Crimson with a 640 nm laser and a 650-760 nm emission filter with 800 ms exposure at 95% laser power.

For fluorescence imaging of PER2-LUC fibroblasts, cells were plated to ~7% confluency in a black, clear-bottomed 96 well plate in high-glucose (27.8 mM), glutamax-containing DMEM (GIBCO) supplemented with 10% serum (HyClone FetalClone III, Themofisher) and pen/strep and maintained under temperature cycles as described above. After 24 h, they were treated with Cell Tracker Red CMTPX Dye (Thermo Fisher) for 30 min before changing to MOPS-buffered ‘Air medium’ with 10% serum but lacking luciferin and moving to an Opera Phenix Plus (Revvity Perkin Elmer, at the UCSC Chemical Screening Center RRID SCR_021114)) for recording at constant 37 °C in a humidified environment. Cells were imaged every 15 min using the same hardware as described above for Rev-Erbα-Venus FUCCI-2A 3T3s, except the focal plane was at -8 um and the following channels: brightfield with transmitted light and a 650-760 nm filter with 100 ms exposure at 40% light power; Cell Tracker Red with a 561 nm excitation laser and a 570-630 nm emission filter with 100 ms exposure at 95% laser power.

Fluorescence image analysis of 3T3s and primary fibroblasts was performed using the cell tracking feature of Harmony 5.1 high-content imaging and analysis software (Revvity Perkin Elmer, at the UCSC Chemical Screening Center (RRID SCR_021114)). For Rev-Erbα-Venus FUCCI-2A 3T3s, the images were flatfield corrected, the sum of the three fluorescent channels calculated, and a sliding parabola applied, from which nuclei were identified, nuclei intersecting with the image border removed, the nuclei then tracked (with tracked nuclei requiring an overlap of at least 1% between sequential images), and the intensities of the three fluorescent channels measured for each nucleus. For subsequent analysis, only cells with continuous tracks longer than 200 images were used. Senescent cells were defined as those cells that did not undergo a cell division event during the recording, determined as those cells which did not complete a cycle of cdt1 and geminin expression from the FUCCI-2A reporter. Period of cell division was determined from those cells that underwent at least two full cycles of cell-cycle gene expression, with period quantification performed using BioDare 2^60^. Relative timing of cdt1 peak expression to geminin expression was determined by manually identifying the peak of cdt1 expression relative to the trough and peak of the concurrent cycle of geminin expression. This manual analysis was performed blind.

For Cell Tracker Red images, PER2-LUC fibroblasts were tracked as described above for the 3T3s except the Cell Tracker Red raw image was used for cell identification, and the number of cells was outputted.

### Statistics

All statistical analysis was performed using Prism 10 (GraphPad). All replicates are biological replicates. Sample sizes were based on field-specific standards.

### Reporting summary

Further information on research design is available in the Nature Portfolio Reporting Summary linked to this article.

## Supplementary information

Supplementary Information

Transparent Peer Review file

Reporting Summary

Description of Additional Supplementary Files

Supplementary Movie 1

Supplementary Movie 2

Supplementary Movie 3

## Source data

Source Data

## Data Availability

Source data are provided as a Source Data folder. The data are available upon request. Source data are provided with this paper.
