# Supplementary material for: Intracellular potassium levels orchestrate circadian rhythmicity and cell division
Source: Nat Commun. 2026 May 22;17:6738. doi: 10.1038/s41467-026-73351-2 (PMC13385874; doi:10.1038/s41467-026-73351-2)
Supplement: Supplementary file 1 — Supplementary Information [file 41467_2026_73351_MOESM1_ESM.pdf]

SUPPLEMENTARY INFORMATION

Intracellular potassium levels orchestrate circadian rhythmicity and cell division.

Sergio Gil Rodríguez, Louise L. Hansen, Olivia J. P. Fraser, Yen Peng Chew, Rebecca K. Spangler, Ellen Grünewald, Andrew D. Beale, Beverley M. Rabbitts, Alessandra Stangherlin, John S. O'Neill, Carrie L. Partch, Priya Crosby, Gerben van Ooijen

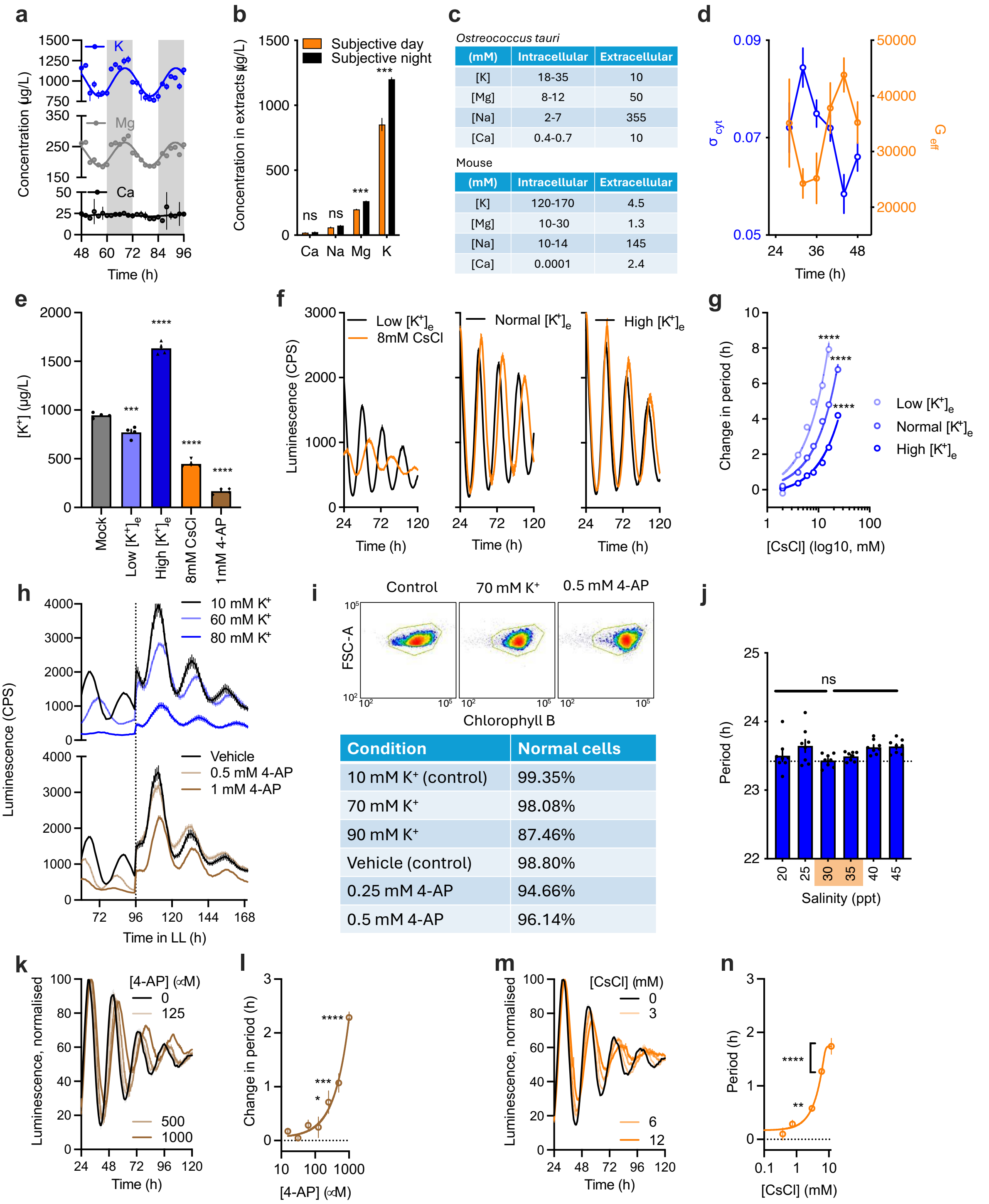

Ostreococcus tauri

(mM)	Intracellular	Extracellular
[K]	18-35	10
[Mg]	8-12	50
[Na]	2-7	355
[Ca]	0.4-0.7	10

Mouse

(mM)	Intracellular	Extracellular
[K]	120-170	4.5
[Mg]	10-30	1.3
[Na]	10-14	145
[Ca]	0.0001	2.4

Condition	Normal cells
10 mM K⁺ (control)	99.35%
70 mM K⁺	98.08%
90 mM K⁺	87.46%
Vehicle (control)	98.80%
0.25 mM 4-AP	94.66%
0.5 mM 4-AP	96.14%

Supplementary Figure 1. Intracellular potassium levels regulate circadian gene expression. Panels a-b, d-j contain *Ostreococcus* data, panels k-n contain mouse fibroblast data. **a)** Quantification of intracellular

potassium, magnesium, and calcium concentrations in *Ostreococcus* cell extracts taken over a time series under constant light conditions. Line represents a sine wave fitted through data points. n=4, mean±SEM. **b)** Quantification of intracellular ions in *Ostreococcus* at subjective day versus subjective night in constant light conditions. n=4, mean±SEM, unpaired t test. **c)** Ion concentrations in *Ostreococcus* extracts were converted to intracellular concentration by correcting dilution rate, total cell number in each sample, and average cell volume. Previously published data[18] from mammalian cells are provided for comparison. **d)** Dielectrophoresis data show rhythmicity in the electophysiological properties of *Ostreococcus* cell (σ_{cyt} = cytoplasmic conductivity; G_{eff} = effective membrane conductance). Experiments were performed under darkness. n=3 to 8 (see source data), mean±SEM. **e)** Changes in intracellular potassium levels can be induced by exogenous treatments in *Ostreococcus*. Samples collected after 16-18h of treatment. n=4, mean±SEM, one-way ANOVA, Dunnett's multiple comparisons test vs. mock. **f-g**) Example traces (f) and dose response curves (g) for the effect of caesium on *Ostreococcus* circadian period in media containing normal (10 mM), low (1 mM), or high (30 mM) extracellular potassium. n=8, mean±SEM, two-way ANOVA, caesium factor 72.2% of variation, Tukey's multiple comparisons test. Data for control media (10mM) is duplicated from main Figure 1c-d). **h)** Control experiments where various concentrations of extracellular potassium (top panel) or 4-AP treatments (bottom panel) were released after 96h, showing a rapid recovery of robust circadian gene expression in *Ostreococcus*. n=16, mean±SEM. **i)** Flow cytometry analyses showing that treatments with high potassium or 4-AP do not affect cell viability in a large majority of *Ostreococcus* cells. **j)** Circadian period of clock gene expression in *Ostreococcus* is robust against overall salinity of the media. All treatments in this manuscript stay within the 30-35 ppt range. n=8, mean±SEM. **k-l)** Example traces (k) and dose response curve (l) of actively dividing PER2-LUC fibroblasts treated with increasing concentrations of 4-AP show increasing circadian period. n=3 or 2 (see source data), mean±SEM, one-way ANOVA, Dunnett's multiple comparisons test vs. mock **m-n)** Example traces (m) and dose response curve (n) of actively dividing PER2-LUC fibroblasts treated with increasing concentrations of CsCl show increasing circadian period. n=3, mean±SEM, one-way ANOVA, Dunnett's multiple comparisons test vs. mock.

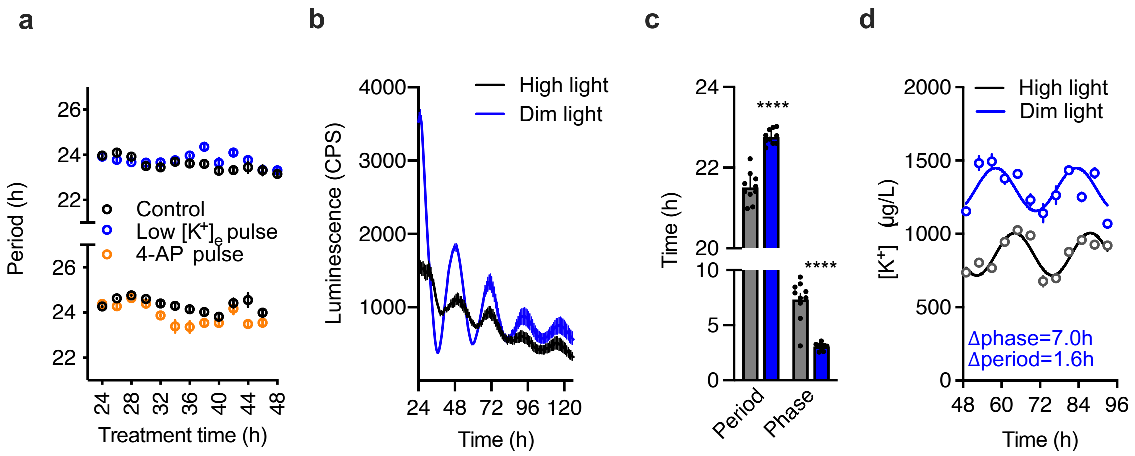

Supplementary Figure 2. Reciprocal interactions between clock gene expression and potassium levels. This figure only contains *Ostreococcus* data. **a)** Quantification of circadian period following the 2h pulsed treatments with 4-AP or low extracellular potassium in main Figure 2d. n=8 to 12 (see source data), mean±SEM. **b)** Traces of the CCA1-LUC marker in high light (black trace) versus dim light (blue trace). High light=16 µmoles/m^2/sec; dim light=2 µmoles/m^2/sec. n=10, mean±SEM. **c)** Quantification of period and phase of the traces in (b). Unpaired t-test. **d)** Quantification of intracellular potassium levels in extracts taken over a time series under constant high or dim light conditions. Line shows sine wave fitted through data points. n=3, mean±SEM.

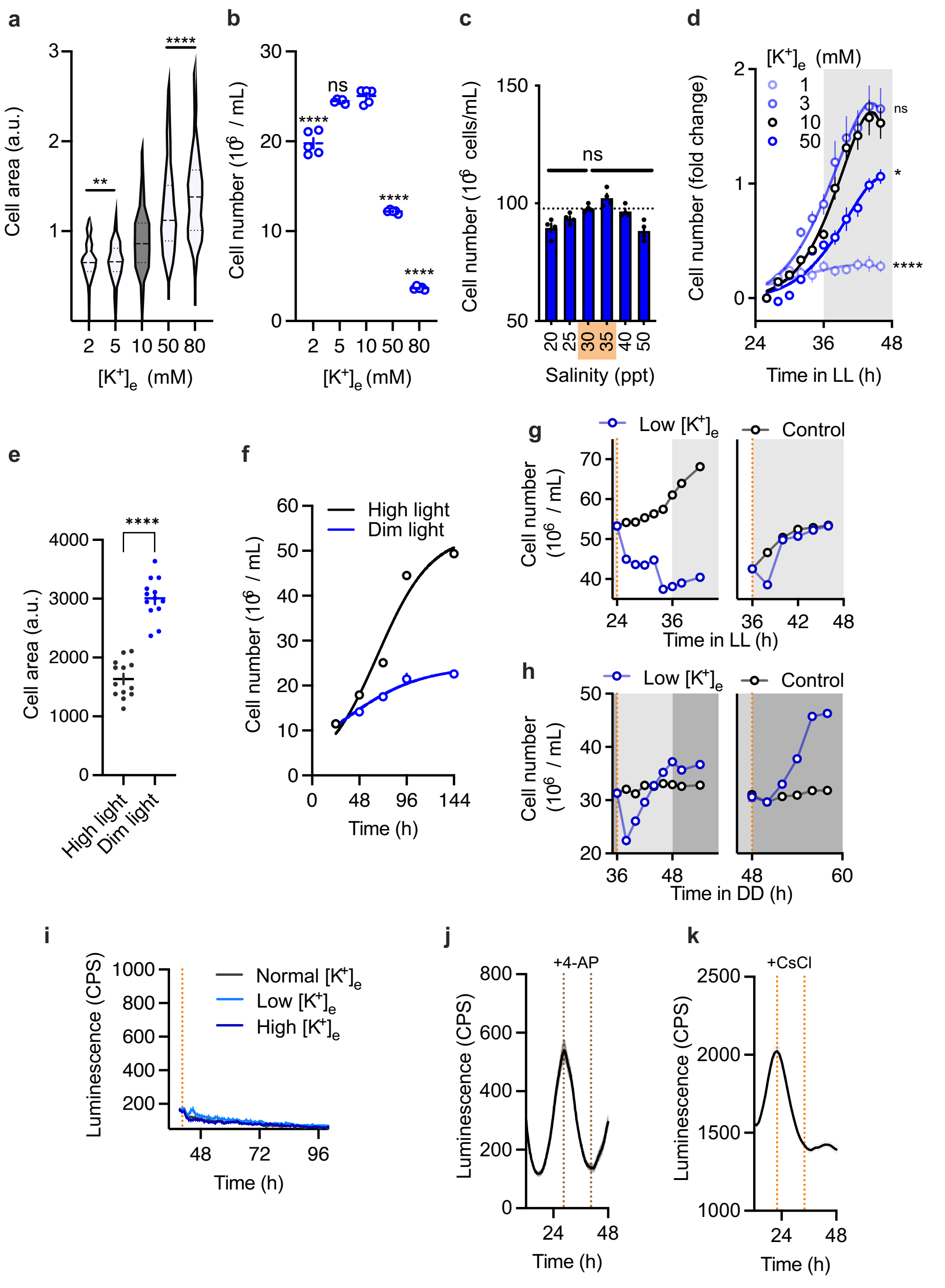

Supplementary Figure 3. Potassium affects cell proliferation. Data in panels a-i was obtained in *Ostreococcus* cells expressing the CCA1-LUC reporter, and data in panels j-k was obtained in mouse fibroblasts expressing PER2-LUC. **a)** High $[K^+]_e$ leads to unusually large cells and low $[K^+]_e$ to unusually small cells. n=50, Mean±SEM, one-way ANOVA, Dunnett's multiple comparisons test vs. 10 mM K^+ control. **b)** Both high and low $[K^+]_e$ lead to lower proliferation rates compared to the standard media containing 10 mM potassium. n=5, Mean±SEM, one-way ANOVA, Dunnett's multiple comparisons test. **c)** The effects in (b) are specific to changes in potassium, as changes in overall salinity do not elicit a similar effect. All treatments in this paper lie in a range of 30-35ppt. n=4, mean±SEM. **d)** Changing $[K^+]_e$ leads to a reduction or even a full arrest of cell proliferation. n=10, mean±SEM, Dunnett's multiple comparison test vs 10 mM K^+ control. Data normalised to starting density. Grey area represents subjective night. Curve fits used a beta growth model. **e)** Quantification of cell size under high versus dim light. n=13, mean±SEM, unpaired t test. **f)** Quantification of cell proliferation under high versus dim light. n=5, mean±SEM. **g)** Cell number upon 2h

pulsed treatments with low potassium at subjective dawn (left panels) versus subjective dusk (right panels) under constant light conditions. n=6, mean±SEM. **h)** As (g), but under constant darkness. In (g-h), darker areas represent subjective night and lighter areas subjective day; orange dotted lines indicate treatment times. n=6, mean±SEM. **i)** Luminescent imaging of CCA1-LUC cells subjected to 2h pulses of low/high potassium under darkness. n=9, mean±SEM. **j-k)** Control traces of PER2-LUC fibroblasts to identify the appropriate treatment times for 4-AP (j) and CsCl (k) to generate the data in main Figure 3d-g. n=4, mean±SEM.

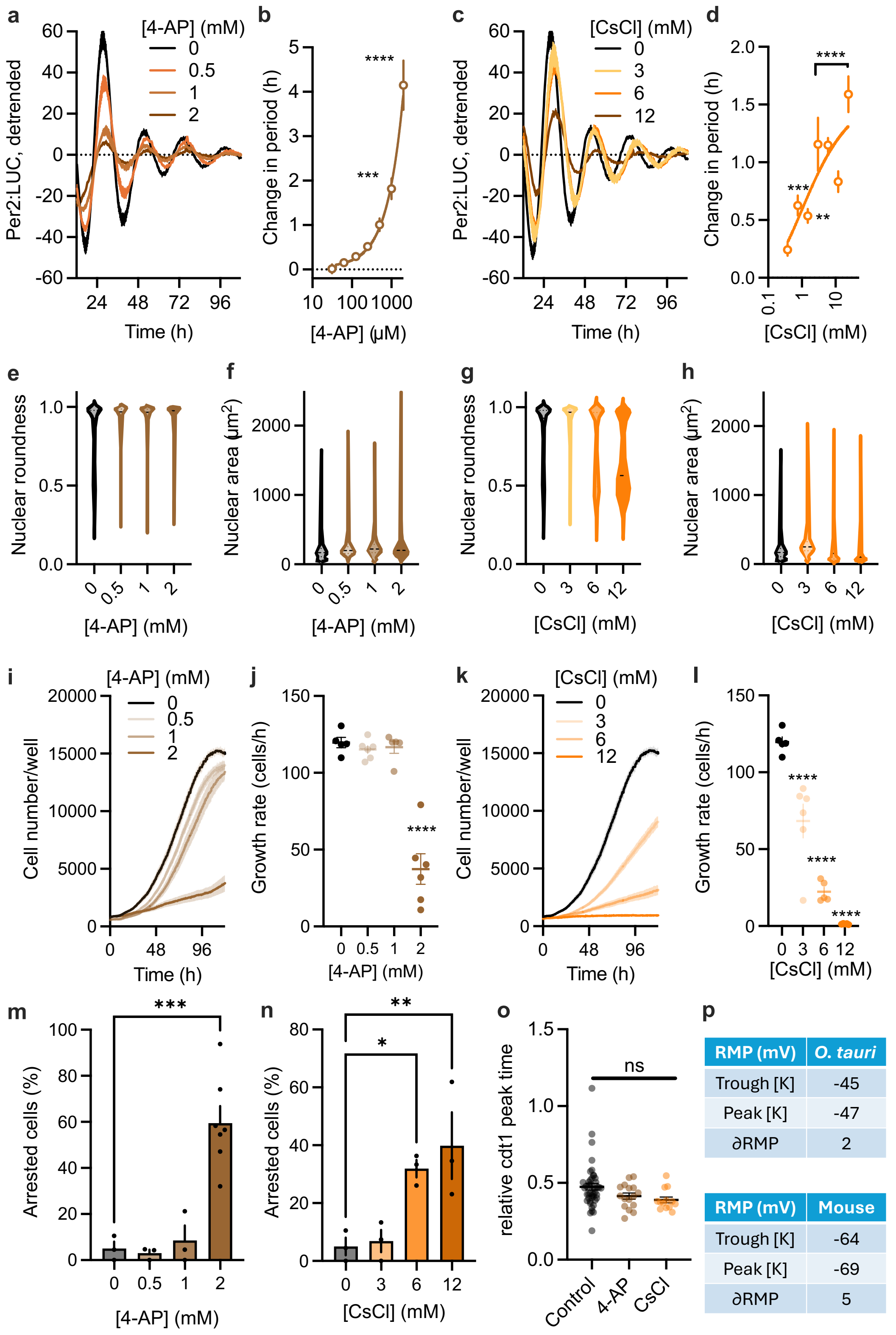

RMP (mV)	*O. tauri*
Trough [K]	-45
Peak [K]	-47
∂RMP	2

RMP (mV)	Mouse
Trough [K]	-64
Peak [K]	-69
∂RMP	5

Supplementary Figure 4. Potassium affects circadian period and cell proliferation in NIH 3T3 cells. This figure only contains data obtained in NIH 3T3 cells. **a-d)** Actively dividing Per2:LUC NIH 3T3 cells treated with increasing concentrations of 4-AP (a-b) or CsCl (c-d) show increasing circadian period. n=4 or 3 (see source data), mean±SEM, one-way ANOVA, Dunnett's multiple comparisons test vs. control. **e-h)** Analysis of the size and shape of nuclei from NIH 3T3 FUCCI cells upon treatment with 4-AP (e-f) or CsCl (g-h). n=14500 or more nuclei (see source data). **i-j)** Treatment of phase-unsynchronised FUCCI NIH 3T3s with 4-AP reduces log-phase proliferation rate in a dose-dependent manner. n=4 to 6 (see source data),

mean±SEM, Dunnett's multiple comparisons test. **k-l)** Treatment of phase-unsynchronised FUCCI NIH 3T3s with CsCl reduces log-phase proliferation rate in a dose-dependent manner. n≥4, mean±SEM, Dunnett's multiple comparisons test. **m-n)** Percentage of cells that failed to complete a division event under increasing concentrations of 4-AP (m) or CsCl (n). n=3 wells, mean±SEM, Dunnett's multiple comparisons test. **o)** The timing of the peak of Cdt1 expression against the trough (0) to peak (1) of geminin expression show no significant difference between the control and cells treated with 12 mM CsCl or 2 mM 4-AP. n=13 to 45 (see source data), mean±SEM, one-way ANOVA, Dunnett's multiple comparisons test. **p)** Resting Membrane Potential (RMP) at peak and trough levels of potassium were calculated over the circadian cycle using the Goldman-Hodgkin-Katz Equation and the values in Supplementary Figure 1. We assumed permeability to these ions to be constant over time. For *Ostreococcus*, we used chloride values of $[Cl^-]_o$ = 385 mM and $[Cl^-]_i$ = 10 mM. For mammalian cells, we used previously published data[18].

.

SUPPLEMENTARY FILES

Supplementary Movie 1

Supplementary Movie 2

Supplementary Movie 3
