# Supplementary material for: Intracellular potassium levels orchestrate circadian rhythmicity and cell division
Source: Nat Commun. 2026 May 22;17:6738. doi: 10.1038/s41467-026-73351-2 (PMC13385874; doi:10.1038/s41467-026-73351-2)
Supplement: Supplementary file 3 — Reporting Summary [file 41467_2026_73351_MOESM3_ESM.pdf]

nature portfolio

Corresponding author(s): Gerben van Ooijen ; Priya Crosby

Last updated by author(s): Apr 17, 2026

Reporting Summary

Nature Portfolio wishes to improve the reproducibility of the work that we publish. This form provides structure for consistency and transparency in reporting. For further information on Nature Portfolio policies, see our Editorial Policies and the Editorial Policy Checklist.

Statistics

For all statistical analyses, confirm that the following items are present in the figure legend, table legend, main text, or Methods section.

n/a	Confirmed	
☐	☒	The exact sample size (*n*) for each experimental group/condition, given as a discrete number and unit of measurement
☐	☒	A statement on whether measurements were taken from distinct samples or whether the same sample was measured repeatedly
☐	☒	The statistical test(s) used AND whether they are one- or two-sided<br>*Only common tests should be described solely by name; describe more complex techniques in the Methods section.*
☐	☒	A description of all covariates tested
☐	☒	A description of any assumptions or corrections, such as tests of normality and adjustment for multiple comparisons
☐	☒	A full description of the statistical parameters including central tendency (e.g. means) or other basic estimates (e.g. regression coefficient) AND variation (e.g. standard deviation) or associated estimates of uncertainty (e.g. confidence intervals)
☐	☒	For null hypothesis testing, the test statistic (e.g. *F*, *t*, *r*) with confidence intervals, effect sizes, degrees of freedom and *P* value noted<br>*Give P values as exact values whenever suitable.*
☒	☐	For Bayesian analysis, information on the choice of priors and Markov chain Monte Carlo settings
☒	☐	For hierarchical and complex designs, identification of the appropriate level for tests and full reporting of outcomes
☒	☐	Estimates of effect sizes (e.g. Cohen's *d*, Pearson's *r*), indicating how they were calculated

Our web collection on statistics for biologists contains articles on many of the points above.

Software and code

Policy information about availability of computer code

Data collection	Cellular fluorescence imaging of mammalian cells was performed using Harmony 5.1 high-content imaging and analysis software (Revvity Perkin Elmer). Longitudinal bioluminescence of mammalian cells was collecting using LumiCycle Analysis Software (Actimetrics) or Andor Solis 64-bit (Andor). Bioluminescence of algal cells was collected on a TriStar2 plate reader (Berthold) using ICE software (Berthold).
Data analysis	Analysis of cellular fluorescence imaging of mammalian cells was performed using Harmony 5.1 high-content imaging and analysis software (Revvity Perkin Elmer). Statistical analysis was performed using GraphPad Prism 10.

For manuscripts utilizing custom algorithms or software that are central to the research but not yet described in published literature, software must be made available to editors and reviewers. We strongly encourage code deposition in a community repository (e.g. GitHub). See the Nature Portfolio guidelines for submitting code & software for further information.

Data

Policy information about availability of data

All manuscripts must include a data availability statement. This statement should provide the following information, where applicable:

- Accession codes, unique identifiers, or web links for publicly available datasets
- A description of any restrictions on data availability
- For clinical datasets or third party data, please ensure that the statement adheres to our policy

Source data are provided as a Source Data folder. The data are available upon request.

Research involving human participants, their data, or biological material

Policy information about studies with human participants or human data. See also policy information about sex, gender (identity/presentation), and sexual orientation and race, ethnicity and racism.

Reporting on sex and gender	n/a
Reporting on race, ethnicity, or other socially relevant groupings	n/a
Population characteristics	n/a
Recruitment	n/a
Ethics oversight	n/a

Note that full information on the approval of the study protocol must also be provided in the manuscript.

Field-specific reporting

Please select the one below that is the best fit for your research. If you are not sure, read the appropriate sections before making your selection.

☒ Life sciences ☐ Behavioural & social sciences ☐ Ecological, evolutionary & environmental sciences

For a reference copy of the document with all sections, see nature.com/documents/nr-reporting-summary-flat.pdf

Life sciences study design

All studies must disclose on these points even when the disclosure is negative.

Sample size	All experiments had a minimum of 3 independent biological replicates, apart from -omics analyses for which biological replication was included in a single experiment.
Data exclusions	Data exclusions made were in mammalian cell proliferation experiments using mouse lung fibroblasts. Wells that had clearly reduced seeding density compared to the rest of the plate, and so a longer intial lag growth phase, were removed to aid data interpretation.
Replication	All experiment were carried out with a minimum of 3 independent biological replicates in order to confirm reproducibility (outlier removal, as above, means a small number of treatments have n=2, and these cases have been highlighted in the figure legends). No experiments presented failed to reproduce in our hands.
Randomization	As all mammalian cell dishes and wells were seeded from the same stock in parallel, and so have been treated identically, randomization is not required.
Blinding	Data collection and analysis was not blinded due to practical difficulties of doing this in cell biology.

Reporting for specific materials, systems and methods

We require information from authors about some types of materials, experimental systems and methods used in many studies. Here, indicate whether each material, system or method listed is relevant to your study. If you are not sure if a list item applies to your research, read the appropriate section before selecting a response.

Materials & experimental systems

n/a	Involved in the study	
☒	☐	Antibodies
☐	☒	Eukaryotic cell lines
☒	☐	Palaeontology and archaeology
☒	☐	Animals and other organisms
☒	☐	Clinical data
☒	☐	Dual use research of concern
☒	☐	Plants

Methods

n/a	Involved in the study	
☒	☐	ChIP-seq
☐	☒	Flow cytometry
☒	☐	MRI-based neuroimaging

Eukaryotic cell lines

Policy information about cell lines and Sex and Gender in Research

Cell line source(s)	Rev-Erbα-Venus FUCCI-2A NIH 3T3s were a gift from Franck Delaunay. PER2::LUC lung fibroblasts were derived from mouse

	lung from a female animal, as described in the methods
Authentication	Cell lines were authenticated through morphology and continued presence of stably expressed reporters.
Mycoplasma contamination	Cells were tested for mycoplasma contamination and found to be negative.
Commonly misidentified lines (See ICLAC register)	None of the commonly misidentified lines were used in this study.

Plants

Seed stocks	n/a
Novel plant genotypes	n/a
Authentication	n/a

Flow Cytometry

Plots

Confirm that:

- [x] The axis labels state the marker and fluorochrome used (e.g. CD4-FITC).
- [x] The axis scales are clearly visible. Include numbers along axes only for bottom left plot of group (a 'group' is an analysis of identical markers).
- [x] All plots are contour plots with outliers or pseudocolor plots.
- [x] A numerical value for number of cells or percentage (with statistics) is provided.

Methodology

Sample preparation	Ostreococcus tauri cells were treated for 120 hours with differential [K+]e or [4-AP] before flow cytometry analyses.
Instrument	BD FACSCanto II (BD Biosciences, San Jose, CA, USA)
Software	BDFacs Diva was used for data acquisition and FCS Express 7 for analysis
Cell population abundance	10,000 cells were analysed per treatment
Gating strategy	Gates were drawn on control samples using forward and side scatter. A further gate was drawn to determine chlorophyll autofluorescence, and 'normal cells' were defined as the percentage of chlorophyll positive cells within sizing gate.

- [x] Tick this box to confirm that a figure exemplifying the gating strategy is provided in the Supplementary Information.
