# Supplementary material for: Intracellular potassium levels orchestrate circadian rhythmicity and cell division
Source: Nat Commun. 2026 May 22;17:6738. doi: 10.1038/s41467-026-73351-2 (PMC13385874; doi:10.1038/s41467-026-73351-2)
Supplement: Supplementary file 4 — Description of Additional Supplementary Files [file 41467_2026_73351_MOESM4_ESM.pdf]

Supplementary Movie 1. Timelapse video of rapidly dividing NIH 3T3 FUCCI-2A cells subjected to single-cell imaging under control conditions.

Supplementary Movie 2. Timelapse video of rapidly dividing NIH 3T3 FUCCI-2A cells subjected to single-cell imaging in the presence of 2 mM 4-AP.

Supplementary Movie 3. Timelapse video of rapidly dividing NIH 3T3 FUCCI-2A cells subjected to single-cell imaging in the presence of 12 mM CsCl.
