# Supplementary material for: Intracellular potassium levels orchestrate circadian rhythmicity and cell division
Source: Nat Commun. 2026 May 22;17:6738. doi: 10.1038/s41467-026-73351-2 (PMC13385874; doi:10.1038/s41467-026-73351-2)
Supplement: Supplementary file 2 — Transparent Peer Review file [file 41467_2026_73351_MOESM2_ESM.pdf]

nature portfolio

# Peer Review File

# Intracellular potassium levels orchestrate circadian rhythmicity and cell division.

Corresponding Author: Professor Gerben van Ooijen

This manuscript has been previously reviewed at another journal. This document only contains information relating to versions considered at Nature Communications.

**This file contains all reviewer reports in order by version, followed by all author rebuttals in order by version.**

Version 0:

Reviewer comments:

Reviewer #1

(Remarks to the Author)
The substantially revised manuscript from Rodriguez and colleagues has addressed all my previous concerns and also is generally much improved thanks to the recommendations of the other reviewers.I feel that it is certainly now suitable for publication in Nature Communications.

Reviewer #2

(Remarks to the Author)
I have no lingering concerns and all issues are addressed.

Reviewer #3

(Remarks to the Author)
The manuscript provides evidence that circadian oscillations in K levels are coupled to cell division; since manipulation of K levels influences the timing of cell division, the authors conclude that there is likely a causal relationship between the oscillations in cytoplasmic K and the timing of division, both in O. taurus and in mammalian cells. My previous objections to the work stemmed mainly from my misunderstanding of several statements. This version of the manuscript is easier to read for non-specialists and, when combined with the rebuttal text, satisfies my concerns. Yet, because potassium is the main factor determining membrane potential in most (if not all) cell types, and certainly in the mammalian cell models used, it would be relevant to discuss to what extent K oscillations drive rhythmicity in cell division because they induce membrane potential oscillations. In other words, are membrane potential oscillations necessary for the appropriate timing of cell division, or is K as such required? With the currently available tools, this should be relatively straightforward to determine (e.g. PMC12715753, PMC12931077). Is the effect observed (which I find convincing) driven by a change in membrane potential?

Minor comment. Mainly in Figure 2 (but also visible in Fig. ED1), there is a clear acute effect of 4AP, low K, or Cs treatment on the reporter activity, CCA1-LUC or PER2-LUC. How do the authors interpret this observation?

**Open Access** This Peer Review File is licensed under a Creative Commons Attribution 4.0 International License, which permits use, sharing, adaptation, distribution and reproduction in any medium or format, as long as you give appropriate credit to the original author(s) and the source, provide a link to the Creative Commons license, and indicate if changes were made.
In cases where reviewers are anonymous, credit should be given to 'Anonymous Referee' and the source.
The images or other third party material in this Peer Review File are included in the article's Creative Commons license, unless indicated otherwise in a credit line to the material. If material is not included in the article's Creative Commons license and your intended use is not permitted by statutory regulation or exceeds the permitted use, you will need to obtain permission directly from the copyright holder.
To view a copy of this license, visit https://creativecommons.org/licenses/by/4.0/

**Reply to reviewers, NCOMMS-26-011915-T**

**We would first like to thank all reviewers for their continued consideration of our manuscript. We very much appreciate your positive response to the substantial improvements we have made to our work. We will reply to your last few remaining comments below, in bold font. Thank you.**

**On behalf of all authors,**
**Priya Crosby and Gerben van Ooijen**
**---**

Reviewer #1:
The substantially revised manuscript from Rodriguez and colleagues has addressed all my previous concerns and also is generally much improved thanks to the recommendations of the other reviewers. I feel that it is certainly now suitable for publication in Nature Communications.

**>>Thank you for your kind comments and your time and effort reviewing this manuscript.**

Reviewer #2:
I have no lingering concerns and all issues are addressed.

**>>We are glad that we were able to address all of your concerns; thank you for your time and effort reviewing this work.**

Reviewer #3:
The manuscript provides evidence that circadian oscillations in K levels are coupled to cell division; since manipulation of K levels influences the timing of cell division, the authors conclude that there is likely a causal relationship between the oscillations in cytoplasmic K and the timing of division, both in O. taurus and in mammalian cells. My previous objections to the work stemmed mainly from my misunderstanding of several statements. This version of the manuscript is easier to read for non-specialists and, when combined with the rebuttal text, satisfies my concerns.

**>>Thank you for your patience - your review helped us greatly in improving our work.**

Yet, because potassium is the main factor determining membrane potential in most (if not all) cell types, and certainly in the mammalian cell models used, it would be relevant to discuss to what extent K oscillations drive rhythmicity in cell division because they induce membrane potential oscillations. In other words, are membrane potential oscillations necessary for the appropriate timing of cell division, or is K as such required? With the currently available tools, this should be relatively straightforward to determine (e.g. PMC12715753, PMC12931077). Is the effect observed (which I find convincing) driven by a change in membrane potential?

**>>Thank you for your additional thoughts. As suggested, we have now discussed the potential contribution of a change in Resting Membrane Potential (RMP) to the effects we observe. Using the concentrations as reported in Supplemental Data Figure 1 and previous work (Stangherlin *et al.*, NComms 2021), we have calculated the changes in RMP at the peak and trough levels of potassium with the Goldman-Hodgkin-Katz Equation. The results are presented in a new Supplemental Data Figure 4p:**

| RMP (mV) | *O. tauri* |
|---|---|
| Trough [K] | -45 |
| Peak [K] | -47 |
| ∂RMP | 2 |

| RMP (mV) | Mouse |
|---|---|
| Trough [K] | -64 |
| Peak [K] | -69 |
| ∂RMP | 5 |

**Additionally, as suggested, we have added a new paragraph to the Discussion section:**

***"As intracellular potassium is the most abundant intracellular cation, the osmotic consequences of oscillations in potassium cannot be separated from concurrent electrochemical changes over the circadian cycle, as evidenced by electrophysiological results in this paper (Extended Data Figure 1) and previous work[21]. From known concentrations of intracellular ions at the peak and trough of potassium levels, we have calculated the difference in Resting Membrane Potential (RMP) over the circadian cycle (Extended Data Figure 4p) and found these to be minor (2 mV for Ostreococcus and 5 mV for mammalian cells). While we do not discount the possibility that changes in RMP make a functional contribution, there is no precedent for such modest changes in RMP to affect cell division. We therefore posit that circadian rhythms in potassium specifically act as a primary factor coupling circadian rhythmicity to the cell cycle."***

**We trust that these changes satisfy the last reviewer comment. Thank you for suggesting it.**

Minor comment. Mainly in Figure 2 (but also visible in Fig. ED1), there is a clear acute effect of 4AP, low K, or Cs treatment on the reporter activity, CCA1-LUC or PER2-LUC. How do the authors interpret this observation?

**>>Thank you. In circadian biology, acute changes in the brightness of luciferase markers upon media changes are commonly observed. These are attributable to transient changes in transport over the plasma membrane of the enzyme substrate, luciferin, which is applied exogenously in the media, and are not due to changes in the abundance of luciferase itself. If the reviewer is interested, one of the laboratories that collaborated on this current study have provided an in-depth analysis of the dynamics of luciferase reporter systems in the Journal of Biological Rhythms in 2016 (PMID: 28112045).**
